# Oxidative Stress-Related Genetic Polymorphisms Are Associated with the Prognosis of Metastatic Gastric Cancer Patients Treated with Epirubicin, Oxaliplatin and 5-Fluorouracil Combination Chemotherapy

**DOI:** 10.1371/journal.pone.0116027

**Published:** 2014-12-29

**Authors:** Ruixuan Geng, Zhiyu Chen, Xiaoying Zhao, Lixin Qiu, Xin Liu, Rujiao Liu, Weijian Guo, Guang He, Jin Li, Xiaodong Zhu

**Affiliations:** 1 Department of Medical Oncology, Fudan University Shanghai Cancer Center, Shanghai, China; 2 Department of Oncology, Shanghai Medical College, Fudan University, Shanghai, China; 3 Bio-X Institutes, Key Laboratory for the Genetics of Developmental and Neuropsychiatric Disorders (Ministry of Education), Shanghai Jiao Tong University, Shanghai, China; Duke Cancer Institute, United States of America

## Abstract

**Background:**

Oxidative stress genes are related to cancer development and treatment response. In this study, we aimed to determine the predictive and prognostic roles of oxidative stress-related genetic polymorphisms in metastatic gastric cancer (MGC) patients treated with chemotherapy.

**Methods:**

In this retrospective study, we genotyped nine oxidative stress-related single nucleotide polymorphisms (SNPs) in *NQO1*, *SOD2*, *SOD3*, *PON1*, *GSTP1*, *GSTT1*, and *NOS3* (rs1800566, rs10517, rs4880, rs1799895, rs662, rs854560, rs1695, rs2266637, rs1799983, respectively) in 108 consecutive MGC patients treated with epirubicin, oxaliplatin, and 5-fluorouracil (EOF) regimen as the first-line chemotherapy and analyzed the association between the genotypes and the disease control rate (DCR), progression-free survival (PFS), and overall survival (OS).

**Results:**

We found that, in addition to a lower pathological grade (*p* = 0.017), *NQO1* rs1800566 CT/TT genotype was an independent predictive factor of poor PFS (hazard ratio [HR] = 1.97, 95% confidence interval [CI] = 1.23–3.16; *p* = 0.005). *PON1* rs662 AA/AG genotype was significantly associated with poor OS (HR = 1.95, 95% CI = 1.07–3.54; *p* = 0.029). No associations were detected between the nine SNPs and DCR.

**Conclusions:**

*NQO1* rs1800566 is an independent predictive factor of PFS for MGC patients treated with EOF chemotherapy, and *PON1* rs662 is a noteworthy prognostic factor of OS. Information on oxidative stress-related genetic variants may facilitate optimization of individualized chemotherapy in clinical practice.

## Background

Gastric cancer is the fourth most common malignant tumors in the world [1]. Metastatic gastric cancer (MGC) has a poor median overall survival (OS) of only 3–5 months when treated with the best supportive care [2]. Systemic chemotherapy is commonly recommended as a fundamental treatment for MGC [3]. Epirubicin, cisplatin, and 5-fluorouracil (5-FU) (ECF) regimen (or its modifications) is a popular and effective first-line treatment for MGC patients. However, only <50% patients are responders and a relative large proportion of patient population do not benefit from this intensive treatment. Therefore, the key issue here is the manner in which to predict the efficacy of chemotherapy and to identify the responders. Thus, it is important to search for convenient-to-use and efficient biomarkers that can predict the response to the therapy and, thereby, the prognosis of the patients.

Oxidative stress plays roles in both carcinogenesis and tumor suppression. The low to moderate levels of reactive oxygen species (ROS) promote tumor growth due to accumulation of mutations, while high dose of ROS cause cancer cell deaths [4]. Epirubicin, oxaliplatin, and 5-FU, the three components of EOF regimen (one type of ECF modifications), generate ROS in their respective metabolic processes, thereby promoting their anticancer effects [5]. Epirubicin intercalates into the DNA and RNA strands, thus prevents synthesis and replication of nucleic acids. ROS generated by epirubicin in mitochondrial respiratory chain or mediated by iron, are regarded as a cause of cardiotoxicity [6]. Oxaliplatin kills cancer cells by formatting platinum-DNA adducts that disrupt DNA replication and transcription by changing the helical structure of DNA. Moreover, the formation of platinum-GSH conjugates results in increased levels of ROS [7]. The anticancer activity of 5-FU is based on its active metabolites, fluorodeoxyuridine triphosphate (FdUTP), fluorodeoxyuridine monophosphate (FdUMP), and fluorouridine triphosphate (FUTP), which inhibit thymidylate synthetase and RNA synthesis [8]. It can also induce mitochondrial ROS generation by a p53-dependent pathway [9]. Antioxidant enzymes such as nicotinamide adenine dinucleotide phosphate (NADPH) quinone oxidoreductase 1 (NQO1), superoxide dismutases (SODs), paraoxonase 1 (PON1), and glutathione S-transferases (GSTs), which are commonly considered as protective agents, may consequently contribute to both body defense and chemotherapy resistance.

NQO1 catalyzes the conversion of quinone to hydroquinone and detoxifies metabolites generated from chemotherapeutic drugs, thus alleviating oxidative stress damage in cells [10]. Recent studies reported association of *NQO1* Pro187Ser polymorphism (rs1800566) with susceptibility to cancers such as gastric and breast cancers [11], [12] as well as with modification of the prognosis of breast cancer treated with anthracycline-based adjuvant chemotherapy [13]. Few studies have been performed on *NQO1* rs10517.

Manganese SOD (MnSOD, SOD2) and extracellular SOD (EcSOD, SOD3) are members of the SOD family, which reduce dismutation of superoxide into oxygen and hydrogen peroxide and thereby prevent cells against oxidative stress. *SOD2* Val16Ala (rs4880) and *SOD3* Arg231Gly (rs1799895) polymorphisms are pathogenic as they induce disturbance in the MnSOD activities and elevate the level of serum EcSOD [14], [15].

PON1 is an antioxidant enzyme that hydrolyses paraoxon [16]. Two common functional genetic polymorphisms, Leu55Met (rs854560) and Gln192Arg (rs662), regulate the serum level and activity of PON [16], [17]. Both the variants are significantly associated with breast and epithelial ovarian cancer vulnerability [18], [19].

GSTs catalyze the conjugation of glutathione to xenobiotics to form glutathione disulfide for the purpose of cell protection [20]. GSTs can also induce drug resistance. Frequent genetic variant of *GSTP1* Ile105Val (rs1695) and *GSTT1*-null have been indicated to be relevant to the response of anthracycline-based chemotherapy in breast cancer patients [21]. Whether the response connects to another common variant Val169Ile in *GSTT1* (rs2266637) remains to be clarified.

In addition to the above-mentioned antioxidant enzymes, an oxidative stress-related gene, *NOS3*, is an oxidative stress inducer as well as an angiogenesis promoter [22]. It encodes endothelial nitric oxide synthase (eNOS), which converts L-arginine, NADPH, and oxygen into nitric oxide (NO) [23]. A polymorphism in *NOS3*, Asp298Glu (rs1799983), has been linked to the risk of cancers such as colon cancer [24] and bladder cancer [25].

This retrospective analysis was performed to investigate the influence of nine oxidative stress-related genetic polymorphisms (rs1800566, rs10517, rs4880, rs1799895, rs662, rs854560, rs1695, rs2266637, rs1799983) toward predicting the response and prognosis of MGC patients treated with the EOF regimen.

## Methods

### Study population

The present retrospective study analyzed the clinical outcomes of 108 consecutive Chinese Han MGC patients treated with EOF as the first-line chemotherapy between May 2009 and June 2012 at the Fudan University Shanghai Cancer Center. All the patients were pathologically diagnosed with gastric cancer and had confirmed metastasis by computed tomography (CT) or magnetic resonance imaging (MRI). Each patient had at least one measurable lesion involved. The patients received EOF treatment comprising of an intravenous infusion of 50 mg/m^2^ epirubicin with a 2-h intravenous infusion of 130 mg/m^2^ oxaliplatin on day 1, followed by a 24-h continuous infusion of 375–425 mg/m^2^/day 5-FU from day 1 to 5. The treatment was given every 3 weeks until disease progression, unacceptable toxicity, or withdrawal at patient’s will or doctor’s discretion. For patients whose lesions continued to shrink after six cycles with good tolerability, 1–2 extra cycles of the treatment were recommended; otherwise, oral FU administration or follow-up was recommended as applicable. The tumor responses were evaluated according to the response evaluation criteria in solid tumors (RECIST) 1.0 guideline every 6 weeks. The patients with complete remission (CR), partial remission (PR), and stable disease (SD) were considered as “controlled.” The patients with progressive disease (PD) were considered as “uncontrolled.” The study was performed in compliance with the principles of the Helsinki Accords and approved by Ethics Committee of Fudan University Shanghai Cancer Center. All patients provided signed informed consent for providing the blood samples to the tissue bank of the Fudan University Shanghai Cancer Center before the start of the treatment.

### Genotyping

We selected and genotyped nine single nucleotide polymorphisms (SNPs) at seven oxidative stress-related genes from NCBI dbSNP database (http://www.ncbi.nlm.nih.gov/SNP/): *NQO1* (rs1800566, rs10517), *SOD2* (rs4880), *SOD3* (rs1799895), *PON1* (rs662, rs854560), *GSTP1* (rs1695), *GSTT1* (rs2266637), and *NOS3* (rs1799983). Genomic DNA was extracted from the whole blood by the standard phenol-chloroform method with AxyPrep Blood Genomic DNA Miniprep Kit (Axygen Scientific, Inc.). All SNPs were genotyped by the TaqMan assay method by using the ABI 7900 DNA Detection System (Applied Biosystems, Foster City, California). All probes and primers were designed by the Assay-on-Design service of Applied Biosystems. The standard polymerase chain reaction (PCR) was performed by using the Taqman Universal PCR Master Mix reagent (Applied Biosystems).

### Statistical analysis

Deviations from the Hardy–Weinberg equilibrium, genotype distributions and allele frequencies, haplotype analysis, and pairwise linkage disequilibrium (LD) were calculated with an online software, SHEsis (http://analysis.bio-x.cn/myAnalysis.php) [26]. The LD of all pairs of SNPs within each gene was tested with D′ and R^2^. Comparisons of the allele and genotype frequencies between the controlled and uncontrolled patients, as well as the relationship between the genotypes and clinicopathological features were based on chi-square or Fisher’s exact probability tests. We used the SPSS software package (version 15.0; SPSS, Chicago, IL, USA) for the analysis of progression-free survival (PFS) and OS by the Kaplan–Meier method and log-rank test. Risk factors at *p*<0.1 were further analyzed as covariates in a multivariate Cox regression model. All *p* values were two-tailed; *p*<0.05 was considered statistically significant.

## Results

### Study population characteristics

Blood samples of all 108 patients were collected before the patients received EOF chemotherapy. Each patient had at least one unresectable lesion, including 36 (33.3%) patients with liver metastasis, 7 (6.5%) with lung metastasis, 31 (28.7%) with ascites, 9 (8.3%) with pleural effusion, and 63 (58.3%) with retroperitoneal lymph node invasion. Among these, 5 (4.6%), 8 (7.4%), and 95 (88.0%) patients had 1, 2, and ≥3 lesions. After EOF treatment, 1 (0.9%), 41 (38.0%), 47 (43.5%), and 19 (17.6%) patients were evaluated as CR, PR, SD, and PD, respectively. 82.4% patients achieved disease control (CR+PR+SD). Age, gender, Eastern Cooperative Oncology Group (ECOG) score, pathological grade, presence/absence of synchronous metastasis, primary lesion, and the number of lesions showed no significant difference between the controlled and uncontrolled groups (*p*>0.05).

### Alleles, genotypes, clinicopathological features and disease control rate (DCR)

Seven oxidative stress-related genes (*NQO1*, *SOD2*, *SOD3*, *PON1*, *GSTP1*, *GSTT1*, *NOS3*) and nine SNPs (rs1800566, rs10517, rs4880, rs1799895, rs662, rs854560, rs1695, rs2266637, rs1799983) analyzed in our study are listed under Table 1. Eight SNPs were located in the coding regions, except rs10517 in the 3′ untranslated region (UTR). The minor allele frequency (MAF) of each SNP in the Han population was >0.03 according to the HapMap database (lack of data: rs1799895 and rs2266637). The Hardy–Weinberg equilibrium of these SNPs was tested in all patients. *SOD3* (rs1799895), *GSTT1* (rs2266637), and *NOS3* (rs1799983) deviated at *p*<0.05 level of significance. Patients with non-informative genotyping for some SNPs (e.g., 4 out of 108 patients for rs1799983) were excluded in corresponding SNP analysis. No allele or genotype showed significant association with DCR (Table 2). We found that there were associations between pathological grade and *GSTP1* rs1695 in codominant model, as well as number of lesions and *SOD3* rs1799895 (*p* = 0.03 and *p* = 0.007, respectively, S1 Table).

**Table 1:** Nine SNPs in the seven oxidative stress-related genes analyzed in the study.

Gene	SNP ID	Location	Alleles	Function	MAF (HCB)*	HWE test P-value**
*NQO1*	rs1800566	chr16∶69745145	T>C	Missense(P187S)	0.478	0.835
	rs10517	chr16∶69743760	C>T	3′UTR	0.381	0.304
*SOD2*	rs4880	chr6∶160113872	T>C	Missense(V16A)	0.146	0.703
*SOD3*	rs1799895	chr4∶24801834	C>G	Missense(R231G)	NA	0.007
*PON1*	rs662	chr7∶94937446	G>A	Missense(Q192R)	0.430	0.678
	rs854560	chr7∶94946084	A>T	Missense(L55M)	0.033	0.727
*GSTP1*	rs1695	chr11∶67352689	A>G	Missense(I105V)	0.207	0.252
*GSTT1*	rs2266637	chr22∶24376845	G>A	Missense(V169I)	NA	4.69e-006
*NOS3*	rs1799983	chr7∶150696111	G>T	Missense(D298E)	0.111	0.021

*HCB: Han Chinese Beijing. NA: Not available in dbSNP.

**HWE: Hardy-Weinberg equilibrium. HWE is tested in all patients.

**Table 2:** Allele and genotype distribution in controlled and uncontrolled patients.

SNP ID	Genotype frequency*			*P-*value**	Allele frequency*		X^2^	*P-*value**	Odds Ratio (95%CI)
**rs1800566**	CC	CT	TT		C	T			
controlled	32(0.360)	41(0.461)	16(0.180)	0.269	105(0.590)	73(0.410)	0.226	0.634	1.19(0.58∼2.46)
uncontrolled	6(0.316)	12(0.632)	1(0.053)		24(0.632)	14(0.368)			
**rs10517**	CC	CT	TT		C	T			
controlled	32(0.364)	39(0.443)	17(0.193)	0.922	103(0.585)	73(0.415)	0.082	0.775	0.90(0.43∼1.88)
uncontrolled	6(0.353)	7(0.412)	4(0.235)		19(0.559)	15(0.441)			
**rs4880**	CC	CT	TT		C	T			
controlled	2(0.023)	15(0.170)	71(0.807)	0.325	19(0.108)	157(0.892)	1.702	0.192	1.87(0.72∼4.82)
uncontrolled	0(0.000)	7(0.368)	12(0.632)		7(0.184)	31(0.816)			
**rs1799895**	CC	CG	GG		C	G			
controlled	83(0.943)	4(0.045)	1(0.011)	0.890	170(0.966)	6(0.034)	0.060	0.807	0.31(0.15∼11.17)
uncontrolled	18(0.947)	1(0.053)	0(0.000)		37(0.974)	1(0.026)			
**rs662**	AA	AG	GG		A	G			
controlled	14(0.165)	38(0.447)	33(0.388)	0.434	66(0.388)	104(0.612)	1.557	0.212	0.61(0.27∼1.34)
uncontrolled	1(0.056)	8(0.444)	9(0.500)		10(0.278)	26(0.722)			
**rs854560**	AA	AT	TT		A	T			
controlled	82(0.921)	7(0.079)	0(0.000)	0.218	171(0.961)	7(0.039)	1.464	0.226	–***
uncontrolled	18(1.000)	0(0.000)	0(0.000)		36(1.000)	0(0.000)			
**rs1695**	AA	AG	GG		A	G			
controlled	64(0.719)	24(0.270)	1(0.011)	0.553	152(0.854)	26(0.146)	0.537	0.464	0.71(0.28∼1.79)
uncontrolled	11(0.611)	7(0.389)	0(0.000)		29(0.806)	7(0.194)			
**rs2266637**	AA	AG	GG		A	G			
controlled	1(0.014)	2(0.028)	68(0.958)	0.689	4(0.028)	138(0.972)	0.980	0.322	–***
uncontrolled	0(0.000)	0(0.000)	17(1.000)		0(0.000)	34(1.000)			
**rs1799983**	GG	GT	TT		G	T			
controlled	69(0.802)	14(0.163)	3(0.035)	0.915	152(0.884)	20(0.116)	0.144	0.704	0.82(0.28∼2.34)
uncontrolled	14(0.778)	3(0.167)	1(0.056)		31(0.861)	5(0.139)			

*Presented as n (frequency).

**Fisher’s *p*-value for all genotype frequency comparisons between the controlled and uncontrolled patients, as well as allele frequency comparisons for rs854560, rs1799895, rs2266637.

***Odds ratio cannot be calculated for no uncontrolled patient carries T allele of rs854560 or A allele of rs2266637.

### Survival analysis

**Figure 1:**
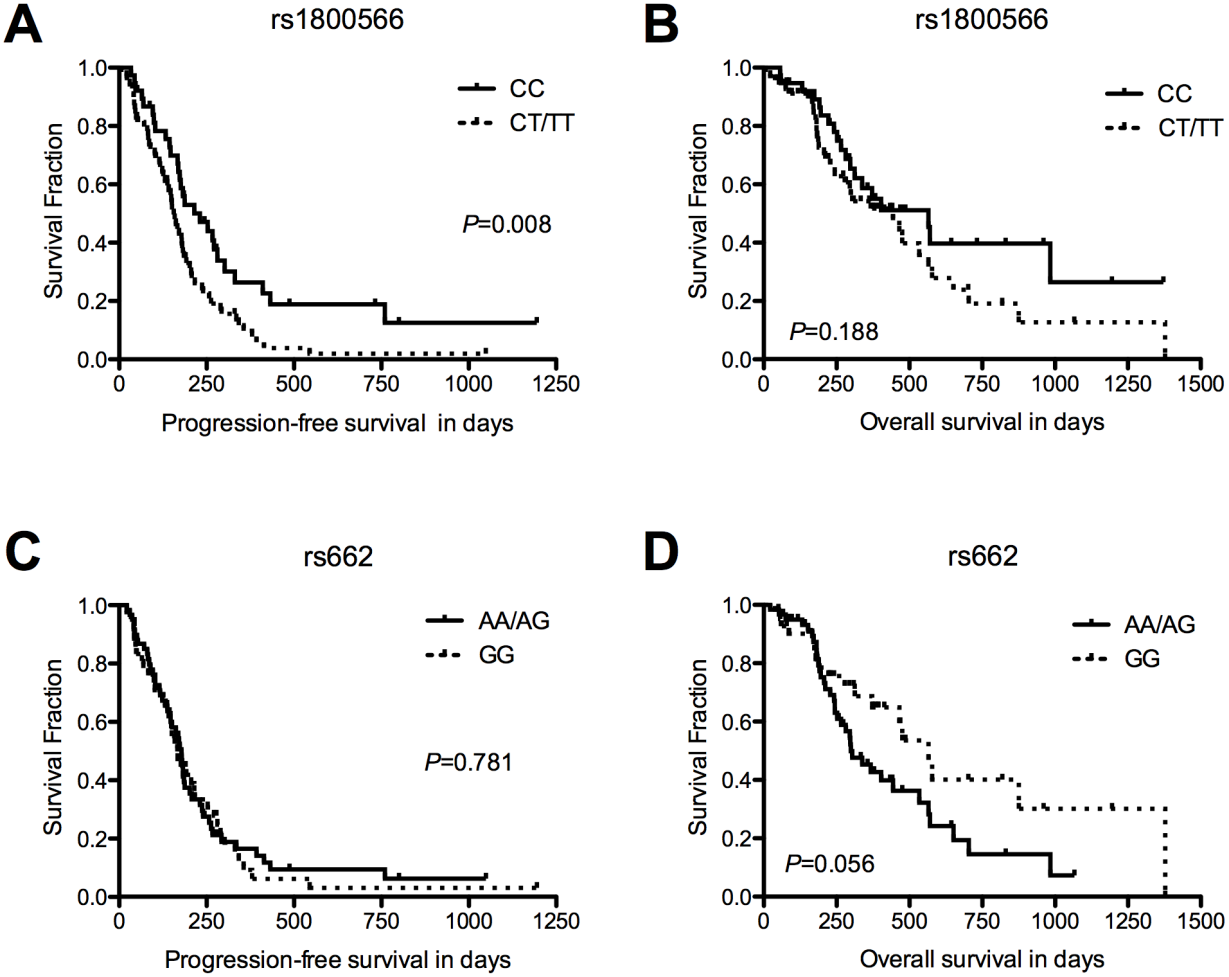
Comparisons of Kaplan-Meier PFS and OS curve between *NQO1* rs1800566 and *PON1* rs662 genotypes among subgroups. **(A, B)** PFS and OS for rs1800566 in dominant model. CT/TT carriers (dotted line, *n* = 70) have a significantly shorter PFS than CC carriers (solid line, *n* = 38). Median PFS for CC and CT/TT genotype: 231.0 *vs.* 156.0 days, *p* = 0.008, log-rank test; median OS: 565.0 *vs.* 444.0 days, *p* = 0.188, log-rank test. **(C, D)** PFS and OS among patients in rs662 genotype subgroups. GG carriers (dotted line, *n* = 42) live significantly longer than AA/AG carriers (solid line, *n* = 61). Median PFS for GG and AA/AG genotype: 166.0 *vs.* 178.0 days, *p* = 0.781, log-rank test; median OS: 565.0 *vs.* 299.0 months, *p* = 0.056, log-rank test.

The Kaplan–Meier analysis with log-rank test of progression-free survival (PFS) indicated that the pathological grade, number of lesions, and genotypes of rs1800566 in *NQO1* were significantly associated with PFS (Table 3). As for rs1800566, patients with CC genotype had a longer median PFS than those carrying CT and TT in both the codominant model (for CC, CT, and TT genotypes; median PFS: 231.0, 159.0, and 149.0 days, respectively; *p* = 0.022) and the dominant model (for CC and CT/TT genotypes; median PFS: 231.0 and 156.0 days, respectively; *p* = 0.008; Fig. 1A). As shown in Table 4, the Cox regression analysis revealed that the patients carrying T allele had an increased risk of disease progression (dominant model, hazard ratio [HR] = 1.97, 95% confidence interval [CI] = 1.23–3.16; *p* = 0.005), demonstrating that, in addition to pathological grade (HR = 0.31, 95% CI = 0.12–0.81; *p* = 0.017), rs1800566 was an independent predictive factor of PFS for patients treated with EOF. No significant association was detected between PFS and the other eight SNPs.

**Table 3:** PFS and OS analysis with Kaplan-Meier method and log-rank test.

Clinical features	Patients (n)	PFS (days)	OS (days)
		Median PFS	95% CI	*P*-Value*	Median OS	95% CI	*P-*Value*
**Age**							
< = 60	80	159.0	129.3–188.7	0.176	465.0	165.5–764.5	0.916
>60	28	206.0	114.5–297.5		403.0	263.4–542.6	
**Gender**							
Male	64	182.0	143.3–220.7	0.163	534.0	314.9–753.1	0.359
Female	44	166.0	141.8–190.2		372.0	245.7–498.3	
**ECOG score** ***							
0	14	240.0	157.2–322.8	0.768	704.0	480.5–927.5	0.153
1	89	167.0	141.3–192.7		367.0	218.3–515.7	
2	5	237.0	0.0–634.2		299.0	0.0–829.3	
**Pathological grade**							
Low and undifferentiated	66	156.0	135.4–176.6	**0.004**	403.0	212.6–593.4	0.226
Moderate and high	13	380.0	178.8–581.2		875.0	170.0–1580.0	
Unclassified	29	180.0	156.1–203.9		367.0	161.8–572.2	
**Synchronous metastasis**							
Presence	88	166.0	143.7–188.3	**0.052**	444.0	295.4–592.6	0.650
Absence	20	253.0	136.5–369.5		565.0	276.9–853.1	
**Primary lesion**							
Cardia	25	180.0	150.5–209.5	0.788	367.0	119.0–614.9	0.626
Non-cardia	83	173.0	150.9–195.1		465.0	267.9–662.1	
**No. of lesions**							
1	5	545.0	117.7–972.3	**0.038**	984.0	–**	**0.076**
2	8	411.0	147.8–674.2		570.0	34.9–1105.1	
3 or more	95	167.0	140.9–193.1		372.0	224.5–519.5	
**rs1800566 (codominant model)**							
CC	38	231.0	133.5–328.5	**0.022**	565.0	320.3–809.7	0.226
CT	53	159.0	132.3–185.7		444.0	236.1–651.9	
TT	17	149.0	94.9–203.1		465.0	164.3–765.7	
**rs1800566 (dominant model)**							
CC	38	231.0	133.5–328.5	**0.008**	565.0	320.3–809.7	0.188
CT+TT	70	156.0	135.9–176.1		444.0	249.2–638.8	
**rs10517**							
CC+CT	84	171.0	147.3–194.7	0.127	444.0	259.5–628.5	0.370
TT	21	267.0	158.7–375.3		565.0	333.8–796.2	
**rs662 (codominant model)**							
AA	15	162.0	118.6–205.4	0.736	265.0	167.4–362.6	**0.087 (AA ** ***vs.*** ** GG: 0.032)**
AG	46	180.0	163.2–196.8		304.0	216.3–391.7	
GG	42	166.0	127.3–204.7		565.0	441.1–688.9	
**rs662 (dominant model)**							
GG	42	166.0	127.3–204.7	0.781	565.0	441.1–688.9	**0.056**
AA+AG	61	178.0	160.5–195.5		299.0	212.2–385.8	

*Factors at *p*<0.1 level enter into Cox regression analysis. *P-*values for further analysis (*p*<0.1) are in bold.

**95% CI cannot be calculated as 3 out of 5 individuals in the subgroup are censored.

***ECOG is one of the first publicly funded cooperative groups to perform multi-center clinical trials for cancer research in USA. ECOG score is a commonly used scoring system for evaluating patients’ performance status.

**Table 4:** Multivariate PFS and OS analysis with Cox regression.

Clinical features	PFS			OS		
	HR	95%CI	*P*-Value*	HR	95%CI	*P*-Value*
**Pathological Grade**						
Low and undifferentiated	1		**0.017** **	–***		
Moderate and high	0.31	0.12–0.81				
Unclassified	0.73	0.44–1.20				
**No. of lesions**						
1	1		0.212	1		0.094
2	0.30	0.06–1.43		2.66	0.27–25.96	
3 or more	0.83	0.26–2.66		6.36	0.83–48.75	
**Synchronous metastasis**						
Presence	1		0.223	–***		
Absence	0.67	0.35–1.28				
**rs1800566**						
CC	1		**0.005**	NA		
CT+TT	1.97	1.23–3.16				
**rs662**						
GG	NA			1		**0.029**
AA+AG				1.95	1.07–3.54	

*Significant *p-*values (*p*<0.05) are in bold.

**P-value is calculated in patients with classified pathological stage.

***Only rs662 and number of lesions are included in the OS analysis based on results in Table 3.

HR: hazard ratio. NA: Not available.

In the Kaplan–Meier analysis of OS, the number of lesions and genotypes of rs662 in *PON1* revealed a borderline significant association with OS (Table 3). Patients with GG genotype had better longevity than AA and AG carriers (for GG and AA/AG genotypes; median OS: 565.0 and 299.0 days, respectively; *p* = 0.056; Fig. 1D). On comparison of AA with GG only, the difference was significant (*p* = 0.032). The number of lesions and rs622 were further entered into the Cox regression model as covariates. The results demonstrated that rs662 was an important prognostic factor of MGC patients treated with EOF (HR = 1.95, 95% CI = 1.07–3.54; *p* = 0.029).

### LD analysis

Pairwise LD between rs1800566 and rs10517 showed D′ = 0.99 and R^2^ = 0.49, indicating no LD between the two SNPs in *NQO1* (S1 Fig.). Haplotype analysis showed that no haplotype of rs1800566 and rs10517 was responsible for disease control (S2 Table).

## Discussion

Oxidative stress is involved in tumor development and in response to systemic therapy. ROS induce mutation in the early stage of cancer as a possible tumor promoter [4]. However, at the advanced stage, ROS have controversial roles: they promote cancer progression with DNA damage and function as intermediates to facilitate chemotherapeutic agents against tumors.

In our study, we examined nine SNPs in seven oxidative stress-related genes: *NQO1*, *SOD2*, *SOD3*, *PON1*, *GSTP1*, *GSTT1*, and *NOS3*. All these genes encode antioxidant enzymes, except *NOS3*, which is a gene encoding eNOS that generates NO in vascular endothelial cells and thereby induces oxidative and nitrosative stress [22], [23]. In addition to the clinical characteristic pathological grade, *NQO1* rs1800566 was found to be an independent predictive factor of PFS for MGC patients treated with EOF regimen. Moreover, we detected a tendency of shorter OS for CT/TT genotype carriers as compared to that of CC genotype, which is similar to our results of PFS analysis (for CC and CT/TT genotypes; median OS: 565.0 and 444.0 days, respectively; *p* = 0.188). Although the difference did not reach a significant level, the shorter tendency of OS, together with the significant poorer PFS of CT/TT genotype carriers indicated a link between T allele and poor treatment outcome.

Our results are supported by those of Fagerholm’s [13], who demonstrated that rs1800566 TT genotype was a poor prognostic and predictive factor in the treatment of breast cancer patients, probably due to the C to T substitution, which causes a Pro to Ser change at the residue 187, resulting in low level of NQO1 activity and impaired detoxification of ROS. Tumor growth is consequently promoted by enhanced genetic instability and antiapoptosis [13]. Fagerholm’s *in vitro* experiments proved that breast cancer cells with TT genotype were resistant to epirubicin, which can be partly attributed to decreased expression of NQO1. This mechanism may also be a potential explanation for the poor treatment outcome of the TT genotype patients treated with EOF in our study.

*NQO1* polymorphisms have been associated with the treatment response in some studies as well. For instance, Barragan [27] and Tian [28] indicated that *NQO1* polymorphism rs1800566 has predictive usefulness toward clinical response to induction therapy (anthracycline- and cytarabine-based regimen) in acute myeloid leukemia (AML) and platinum-based chemotherapy in non-small cell lung cancer (NSCLC) patients, respectively. Both these studies showed that patients with TT genotype have a lower response rate than patients with other genotypes. However, we did not identify the response rate or DCR of EOF treatment in association with the *NQO1* polymorphisms rs1800566 and rs10517. In Barragan’s and Tian’s studies, cisplatin (or carboplatin) is the key component of the doublet regimen in NSCLC and anthracycline is the key factor of the combined regimen in AML. However, in our trial, although the triplet regimen contained oxaliplatin and epirubicin (a type of anthracyclines), 5-FU also played an essential role against cancer progression. Therefore, in MGC patients treated with this triplet EOF regimen, numerous factors are involved in determining the response rate. The role of rs1800566 in predicting the response to the combination regimen in such patients remains unclear.

Compared with PFS, some factors (e.g., second-line or third-line treatment, local therapy) exert their influence on OS besides first-line treatment. In fact, PFS more directly reflects the efficacy of the first-line treatment; therefore, *NQO1* rs1800566 may serve as a surrogate biomarker in predicting the short-term therapeutic effect of EOF triplet regimen in MGC patients. It may also serve as a potential biomarker for selecting patients more likely to benefit from the treatment of EOF regimen.

Another polymorphism of *NQO1*, rs10517, was tested. There exists no published data on the relationship between the clinical outcome of cancer patients and this SNP. We found that TT carriers were likely to have a tendency of better PFS because the survival curve of TT carriers showed a clear trend of deviation from the curve of other patients, but the difference was not significant (for CC/CT and TT genotypes; median PFS: 171.0 and 267.0 days, respectively; *p* = 0.127). Further studies are required to determine whether rs10517, like rs1800566, is also an important biomarker.

Some studies have indicated a link among the PON1 activity, gastric cancer susceptibility, and patients’ OS. It has been shown that the serum arylesterase activity of PON1 was reduced significantly in patients with gastroesophageal cancer than in healthy control [29]. Atay [30] reported that the PON1 activity is an independent predictor of OS for patients with gastric cancer (both metastatic and nonmetastatic). The PON1 serum level and activity, which correspond to oxidative stress and inflammatory response, are affected by *PON1* polymorphisms such as rs854560 and rs662. In detail, a Glu to Arg change at the codon 192 from an A to G substitution in rs662 is correlated with increased PON1 activity, suggesting a potential link between rs662 and gastric cancer.

Consistent with Atay’s study, we identified a notable association between rs662 and OS of MGC patients treated with EOF regimen: AA and AG carriers (HR = 1.95) have a poor median OS as compared with GG carriers. Considering that no relationship has been identified between rs662 and DCR or PFS of EOF treatment, we presume that rs662 could be a prognostic biomarker of OS for MGC patients. Although several studies have attempted to investigate association between *PON1* polymorphisms and cancer susceptibility, to the best of our knowledge, no study has focused on the relationship between *PON1* polymorphisms and chemotherapeutic outcomes including the response rate, PFS, and OS. Thus, this is the first report of prognostic value for rs662 in the treatment of MGC.

Although SODs and GSTs are antioxidant enzymes similar to NQO1 and PON1, studies focusing on the association of the formers with the therapeutic effect of chemotherapy have drawn contradictory conclusions. High SODs have been suggested to have a detrimental effect, instead of a protective effect, in cancer. For example, a study investigating chemotherapy and MnSOD proved that tumor cells adapted to oxidative stress by gaining MnSOD are resistant to 5-FU [31]. Furthermore, C allele in rs4880, which leads to a higher MnSOD antioxidant activity, has lesser treatment-related toxicity and shorter PFS after adjuvant chemotherapy [32]. In contrast, *GSTP1* rs1695 G allele with low GST level indicates an enhanced risk of chemoresistance to palliative chemotherapy in advanced gastric cancer [33]. NOS3, which acts as a probable oxidative stress-inducer, adds evidence to the argument. Choi *et al*. [34] revealed that women with lower NO-encoding genotypes tend to experience disease progression during adjuvant therapy of breast cancer, which support the notion that ROS are essential for inducing chemotherapeutic response. However, our results were not in accordance with the results of Choi *et al*. Therefore, the prognostic or predictive value of these SNPs needs to be further elucidated in future studies with a larger sample size.

Our data suggest that the polymorphisms of *NQO1* and *PON1* are highly related with the clinical outcome of MGC treated with EOF regimen. Both the SNPs can be easily identified before the treatment, thus providing a method to predict the patients’ short-term and long-term efficacy and helping in evaluating the medical choices and in making clinical decisions.

Our study has some limitations. Our sample size was relatively small, and we did not detect all of the polymorphism sites of each target gene as we selected only some sites that were considered important. In addition, we did not set an additional validation set; hence, more clinical data is required for further verification of the data. In future studies, we plan to investigate the underlying mechanism of these SNPs affecting the patient’s chemotherapy response and prognosis in a larger and more intensive set-up.

## Conclusions

Our study reveals that *NQO1* rs1800566 is an independent predictive factor of PFS for MGC patients treated with EOF chemotherapy and that *PON1* rs662 is an important prognostic factor of OS. We believe that our results need to be confirmed in a larger study cohort to confirm the substantial benefit of the treatment regimen to patients carrying a specific genotype. Moreover, genotype-based drug selection has the potential to become more meaningful in the future after validation in a large patient population in order to allow oncologists to personalize the treatments for patients.

## Supporting Information

S1 Fig **Linkage disequilibrium for a haplotype block within rs1800566 and rs10517.** The number in the square is D’*100 between the two SNPs. D’ = 0.99, r^2^ = 0.49.
(DOCX)

S1 Table *P*-values of chi-square test or Fisher’s exact probability test between clinicopathological features and nine SNPs (in codominant model).
(DOCX)

S2 Table Halpotype analysis of rs1800566 and rs10517.
(DOCX)

## References

[1] Jemal A, Bray F, Center MM, Ferlay J, Ward E, et al (2011) Global cancer statistics. CA Cancer J Clin 61:69–90. 21296855 10.3322/caac.20107

[2] Glimelius B, Ekstrom K, Hoffman K, Graf W, Sjoden PO, et al (1997) Randomized comparison between chemotherapy plus best supportive care with best supportive care in advanced gastric cancer. Ann Oncol 8:163–168. 10.1023/a:1008243606668 9093725

[3] Wagner AD, Unverzagt S, Grothe W, Kleber G, Grothey A, et al. (2010) Chemotherapy for advanced gastric cancer. Cochrane Database Syst Rev: CD004064. 10.1002/14651858.CD004064.pub3 20238327

[4] Gorrini C, Harris IS, Mak TW (2013) Modulation of oxidative stress as an anticancer strategy. Nat Rev Drug Discov 12:931–947. 24287781 10.1038/nrd4002

[5] Conklin KA (2004) Chemotherapy-associated oxidative stress: impact on chemotherapeutic effectiveness. Integr Cancer Ther 3:294–300. 15523100 10.1177/1534735404270335

[6] Simunek T, Sterba M, Popelova O, Adamcova M, Hrdina R, et al (2009) Anthracycline-induced cardiotoxicity: overview of studies examining the roles of oxidative stress and free cellular iron. Pharmacol Rep 61:154–171. 19307704 10.1016/s1734-1140(09)70018-0

[7] Jungwirth U, Kowol CR, Keppler BK, Hartinger CG, Berger W, et al (2011) Anticancer activity of metal complexes: involvement of redox processes. Antioxid Redox Signal 15:1085–1127. 21275772 10.1089/ars.2010.3663 PMC3371750

[8] Longley DB, Harkin DP, Johnston PG (2003) 5-fluorouracil: mechanisms of action and clinical strategies. Nat Rev Cancer 3:330–338. 12724731 10.1038/nrc1074

[9] Hwang PM, Bunz F, Yu J, Rago C, Chan TA, et al (2001) Ferredoxin reductase affects p53-dependent, 5-fluorouracil-induced apoptosis in colorectal cancer cells. Nat Med 7:1111–1117. 11590433 10.1038/nm1001-1111 PMC4086305

[10] Chen S, Wu K, Knox R (2000) Structure-function studies of DT-diaphorase (NQO1) and NRH: quinone oxidoreductase (NQO2). Free Radic Biol Med 29:276–284. 11035256 10.1016/s0891-5849(00)00308-7

[11] Lajin B, Alachkar A (2013) The NQO1 polymorphism C609T (Pro187Ser) and cancer susceptibility: a comprehensive meta-analysis. Br J Cancer 109:1325–1337. 23860519 10.1038/bjc.2013.357 PMC3778271

[12] Menzel HJ, Sarmanova J, Soucek P, Berberich R, Grunewald K, et al (2004) Association of NQO1 polymorphism with spontaneous breast cancer in two independent populations. Br J Cancer 90:1989–1994. 15138483 10.1038/sj.bjc.6601779 PMC2410282

[13] Fagerholm R, Hofstetter B, Tommiska J, Aaltonen K, Vrtel R, et al (2008) NAD(P)H:quinone oxidoreductase 1 NQO1*2 genotype (P187S) is a strong prognostic and predictive factor in breast cancer. Nat Genet 40:844–853. 18511948 10.1038/ng.155

[14] Bag A, Bag N (2008) Target sequence polymorphism of human manganese superoxide dismutase gene and its association with cancer risk: a review. Cancer Epidemiol Biomarkers Prev 17:3298–3305. 19064542 10.1158/1055-9965.EPI-08-0235

[15] Sandstrom J, Nilsson P, Karlsson K, Marklund SL (1994) 10-fold increase in human plasma extracellular superoxide dismutase content caused by a mutation in heparin-binding domain. J Biol Chem 269:19163–19166. 8034674

[16] Humbert R, Adler DA, Disteche CM, Hassett C, Omiecinski CJ, et al (1993) The molecular basis of the human serum paraoxonase activity polymorphism. Nat Genet 3:73–76. 8098250 10.1038/ng0193-73

[17] Leviev I, Deakin S, James RW (2001) Decreased stability of the M54 isoform of paraoxonase as a contributory factor to variations in human serum paraoxonase concentrations. J Lipid Res 42:528–535. 11290824

[18] Saadat M (2012) Paraoxonase 1 genetic polymorphisms and susceptibility to breast cancer: a meta-analysis. Cancer Epidemiol 36:e101–103. 22133529 10.1016/j.canep.2011.10.015

[19] Lurie G, Wilkens LR, Thompson PJ, McDuffie KE, Carney ME, et al (2008) Genetic polymorphisms in the Paraoxonase 1 gene and risk of ovarian epithelial carcinoma. Cancer Epidemiol Biomarkers Prev 17:2070–2077. 18708400 10.1158/1055-9965.EPI-08-0145 PMC2729507

[20] Hayes JD, McLellan LI (1999) Glutathione and glutathione-dependent enzymes represent a co-ordinately regulated defence against oxidative stress. Free Radic Res 31:273–300. 10517533 10.1080/10715769900300851

[21] Tulsyan S, Chaturvedi P, Agarwal G, Lal P, Agrawal S, et al (2013) Pharmacogenetic influence of GST polymorphisms on anthracycline-based chemotherapy responses and toxicity in breast cancer patients: a multi-analytical approach. Mol Diagn Ther 17:371–379. 23812950 10.1007/s40291-013-0045-4

[22] Fukumura D, Kashiwagi S, Jain RK (2006) The role of nitric oxide in tumour progression. Nat Rev Cancer 6:521–534. 16794635 10.1038/nrc1910

[23] Alderton WK, Cooper CE, Knowles RG (2001) Nitric oxide synthases: structure, function and inhibition. Biochem J 357:593–615. 11463332 10.1042/0264-6021:3570593 PMC1221991

[24] Jang MJ, Jeon YJ, Kim JW, Chong SY, Hong SP, et al (2013) Association of eNOS polymorphisms (-786T>C, 4a4b, 894G>T) with colorectal cancer susceptibility in the Korean population. Gene 512:275–281. 23137631 10.1016/j.gene.2012.10.032

[25] Verim L, Toptas B, Ozkan NE, Cacina C, Turan S, et al (2013) Possible relation between the NOS3 gene GLU298ASP polymorphism and bladder cancer in Turkey. Asian Pac J Cancer Prev 14:665–668. 23621215 10.7314/apjcp.2013.14.2.665

[26] Shi YY, He L (2005) SHEsis, a powerful software platform for analyses of linkage disequilibrium, haplotype construction, and genetic association at polymorphism loci. Cell Res 15:97–98. 15740637 10.1038/sj.cr.7290272

[27] Barragan E, Collado M, Cervera J, Martin G, Bolufer P, et al (2007) The GST deletions and NQO1*2 polymorphism confers interindividual variability of response to treatment in patients with acute myeloid leukemia. Leuk Res 31:947–953. 17118447 10.1016/j.leukres.2006.10.002

[28] Tian G, Wang M, Xu X (2014) The Role of NQO1 Polymorphisms in the Susceptibility and Chemotherapy Response of Chinese NSCLC Patients. Cell Biochem Biophys. 10.1007/s12013-014-9820-z 24464627

[29] Krzystek-Korpacka M, Boehm D, Matusiewicz M, Diakowska D, Grabowski K, et al (2008) Paraoxonase 1 (PON1) status in gastroesophageal malignancies and associated paraneoplastic syndromes - connection with inflammation. Clin Biochem 41:804–811. 18423402 10.1016/j.clinbiochem.2008.03.012

[30] Atay AE, Kaplan MA, Evliyaoglu O, Ekin N, Isikdogan A (2014) The predictive role of Paraoxonase 1 (PON1) activity on survival in patients with metastatic and nonmetastatic gastric cancer. Clin Ter 165:e1–5. 24589953 10.7471/CT.2014.1663

[31] Hwang IT, Chung YM, Kim JJ, Chung JS, Kim BS, et al (2007) Drug resistance to 5-FU linked to reactive oxygen species modulator 1. Biochem Biophys Res Commun 359:304–310. 17537404 10.1016/j.bbrc.2007.05.088

[32] Yao S, Barlow WE, Albain KS, Choi JY, Zhao H, et al (2010) Manganese superoxide dismutase polymorphism, treatment-related toxicity and disease-free survival in SWOG 8897 clinical trial for breast cancer. Breast Cancer Res Treat 124:433–439. 20309628 10.1007/s10549-010-0840-0 PMC2968705

[33] Ruzzo A, Graziano F, Kawakami K, Watanabe G, Santini D, et al (2006) Pharmacogenetic profiling and clinical outcome of patients with advanced gastric cancer treated with palliative chemotherapy. J Clin Oncol 24:1883–1891. 16622263 10.1200/JCO.2005.04.8322

[34] Choi JY, Barlow WE, Albain KS, Hong CC, Blanco JG, et al (2009) Nitric oxide synthase variants and disease-free survival among treated and untreated breast cancer patients in a Southwest Oncology Group clinical trial. Clin Cancer Res 15:5258–5266. 19671875 10.1158/1078-0432.CCR-09-0685 PMC2745926

