# Supplementary figures and images for: Oxidative Stress-Related Genetic Polymorphisms Are Associated with the Prognosis of Metastatic Gastric Cancer Patients Treated with Epirubicin, Oxaliplatin and 5-Fluorouracil Combination Chemotherapy
Source: PLoS One. 2014 Dec 29;9(12):e116027. doi: 10.1371/journal.pone.0116027 (PMC4278770; doi:10.1371/journal.pone.0116027)

**Fig. S1**


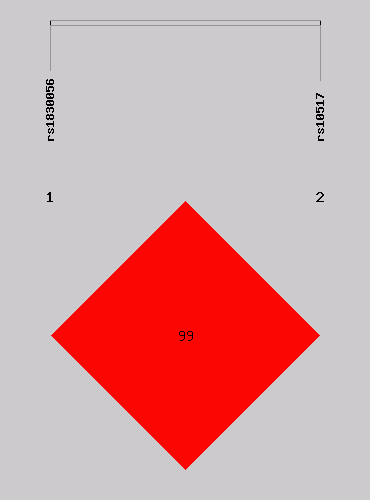


A

Supplement: S1 Fig — Linkage disequilibrium for a haplotype block within rs1800566 and rs10517. The number in the square is D’*100 between the two SNPs. D’ = 0.99, r2 = 0.49. (DOCX) [file pone.0116027.s001.docx]
