# Supplementary material for: Oxidative Stress-Related Genetic Polymorphisms Are Associated with the Prognosis of Metastatic Gastric Cancer Patients Treated with Epirubicin, Oxaliplatin and 5-Fluorouracil Combination Chemotherapy
Source: PLoS One. 2014 Dec 29;9(12):e116027. doi: 10.1371/journal.pone.0116027 (PMC4278770; doi:10.1371/journal.pone.0116027)
Supplement: S1 Table — P-values of chi-square test or Fisher’s exact probability test between clinicopathological features and nine SNPs (in codominant model). (DOCX) [file pone.0116027.s002.docx]

| **Clinicopathological features** | **rs1800566** | **rs10517** | **rs662** | **rs854560** | **rs4880** | **rs1799895** | **rs1695** | **rs2266637** | **rs1799983** |
| --- | --- | --- | --- | --- | --- | --- | --- | --- | --- |
| **Age** | 0.342 | 0.165 | 0.537 | 1.000^**^ | 0.685 | 0.809 | 0.252 | 0.615 | 0.452 |
| **Gender** | 0.544 | 0.836 | 0.611 | 0.236^**^ | 0.599 | 0.701 | 0.141 | 0.156 | 0.245 |
| **ECOG score** | 0.395 | 0.735 | 0.266 | 0.831 | 0.67 | 0.849 | 0.972 | 0.946 | 0.699 |
| **Pathological grade** | 0.917 | 0.788 | 0.843 | 0.344 | 0.915 | 0.442 | **0.03**^*^ | 0.744 | 0.255 |
| **Synchronous metastasis** | 0.833 | 0.871 | 0.155 | 0.348^**^ | 0.664 | 0.89 | 0.614 | 0.097 | 0.116 |
| **Primary lesion** | 0.992 | 0.471 | 0.083 | 1.000^**^ | 0.445 | 0.379 | 0.596 | 0.136 | 0.067 |
| **No. of lesions** | 0.267 | 0.69 | 0.623 | 0.664 | 0.784 | **0.007**^*^ | 0.961 | 0.979 | 0.962 |

**Table S1. *P*-values of chi-square test or Fisher’s exact probability test between clinicopathological features and nine SNPs (in codominant model).**

* Significant *p-*values (*p* < 0.05) are in bold.

** *P*-values of Fisher’s exact probability test.
