# Supplementary material for: Oxidative Stress-Related Genetic Polymorphisms Are Associated with the Prognosis of Metastatic Gastric Cancer Patients Treated with Epirubicin, Oxaliplatin and 5-Fluorouracil Combination Chemotherapy
Source: PLoS One. 2014 Dec 29;9(12):e116027. doi: 10.1371/journal.pone.0116027 (PMC4278770; doi:10.1371/journal.pone.0116027)
Supplement: S2 Table — Halpotype analysis of rs1800566 and rs10517. (DOCX) [file pone.0116027.s003.docx]

**Table S2. Halpotype analysis of rs1800566 and rs10517.**

| **Haplotype** | **Uncontrolled**^*^ | **Controlled**^*^ | **X^2^** | ***P*-Value** | **Odds Ratio (95%CI)** |
| --- | --- | --- | --- | --- | --- |
| C C | 7(0.206) | 30(0.170) | 0.247 | 0.620 | 1.262 (0.503~3.165) |
| C T | 15(0.441) | 73(0.415) | 0.082 | 0.775 | 1.114 (0.531~2.336) |
| T C | 12(0.353) | 73(0.415) | 0.452 | 0.501 | 0.770 (0.358~1.653) |

*Presented as n (frequency)

Frequency<0.03 in both groups has been dropped.
